# Field-Gated Anion Transport in Nanoparticle Superlattices Controlled by Charge Density and Ion Geometry: Insights from Molecular Dynamics Simulations

**DOI:** 10.3390/biom15101427

**Published:** 2025-10-08

**Authors:** Yuexin Su, Jianxiang Huang, Zaixing Yang, Yangwei Jiang, Ruhong Zhou

**Affiliations:** 1 School of Physics, Zhejiang University, Hangzhou 310027, China; 2 Institute of Quantitative Biology, College of Life Sciences, Zhejiang University, Hangzhou 310027, China; 3 Department of Chemistry, Columbia University, New York, NY 10027, USA

**Keywords:** nanoparticle superlattices, ionic conductivity, anion properties, electrostatic gating, molecular dynamics simulations

## Abstract

Nanoparticle superlattices—periodic assemblies of uniformly spaced nanocrystals—bridge the nanoscale precision of individual particles with emergent collective properties akin to those of bulk materials. Recent advances demonstrate that multivalent ions and charged polymers can guide the co-assembly of nanoparticles, imparting electrostatic gating and enabling semiconductor-like behavior. However, the specific roles of anion geometry, valency, and charge density in mediating ion transport remain unclear. Here, we employ coarse-grained molecular dynamics simulations to investigate how applied electric fields (0–0.40 V/nm) modulate ionic conductivity and spatial distribution in trimethylammonium-functionalized gold nanoparticle superlattices assembled with four phosphate anions of distinct geometries and charges. Our results reveal that linear anions outperform ring-shaped analogues in conductivity due to higher charge densities and weaker interfacial binding. Notably, charge density exerts a greater influence on ion mobility than size alone. Under strong fields, anions accumulate at nanoparticle interfaces, where interfacial adsorption and steric constraints suppress transport. In contrast, local migration is governed by geometrical confinement and field strength. Analyses of transition probability and residence time further indicate that the rigidity and delocalized charge of cyclic anions act as mobility barriers. These findings provide mechanistic insights into the structure–function relationship governing ion transport in superlattices, offering guidance for designing next-generation ion conductors, electrochemical sensors, and energy storage materials through anion engineering.

## 1. Introduction

Nanoparticle (NP) superlattices—periodic arrays of nanocrystals—resemble crystalline solids in their structural regularity, yet exhibit emergent collective properties that transcend those of their individual building blocks [1]. These assemblies typically form through the self-assembly of surface-functionalized NPs, guided by a complex interplay of van der Waals (vdW) forces, electrostatic attractions, entropic effects, and ligand-mediated interactions [2,3,4,5]. The resulting long-range order and interparticle coupling endow superlattices with highly tunable electronic [6], optical [7], and magnetic [8] functionalities, positioning them as a versatile platform for various applications, ranging from nanoelectronics, photonics, catalysis, to sensing [9,10,11].

Recent advances have introduced a new paradigm for constructing such materials: electrostatically driven co-assembly involving charged NPs and multivalent counterions or polymers [12,13,14]. For example, Bian et al. [13] demonstrated that trivalent and higher-charged anions—including trimetaphosphate (denoted P3^3−^), pyrophosphate (P2^4−^), and hexametaphosphate (P6^6−^)—can mediate the aqueous co-assembly of oppositely charged NPs. These anions not only bridge NPs via electrostatic interactions but also govern interparticle spacing and superlattice symmetry. Similarly, Thrasher et al. [14] leveraged multivalent polymers to engineer supramolecular interfaces with enhanced architectural complexity. Different from the traditional assembly strategies that rely primarily on ligand design, ion- and polymer-mediated approaches introduce an orthogonal axis of tunability, significantly broadening the accessible design space of NP superlattices [15].

Of particular interest is the emergence of semiconductor-like behavior in such superlattices [16,17]. Coarse-grained molecular dynamics (CGMD) simulations by Lionello et al. [16] revealed that citrate-mediated gold NP superlattices, functionalized with positively charged ligands, can form face-centered cubic (FCC) structures that support field-induced ionic transport. Under applied electric fields, mobile counterions undergo dynamic redistribution, giving rise to electrostatic gating and emergent conductive behavior. Despite this progress, the mechanistic influence of ion-specific features—such as valency, geometry, and charge density—on charge transport remains poorly understood.

Here, we address this knowledge gap using CGMD simulations to examine field-driven anion transport in superlattices composed of trimethyl (mercaptoundecyl) ammonium (TMA)-functionalized gold NPs. We focus on four representative phosphate anions: P2^4−^, P3^3−^, P4^6−^ (tetraphosphate), and P6^6−^, which differ in structure and charge. Our results uncover how anion geometry and charge density modulate ionic conductivity, spatial localization, and dynamic exchange between lattice compartments, establishing key design rules for tailoring ion transport in nanostructured materials.

## 2. Models and Methods

The CG models for the phosphate anions, which follow closely with the experimental work reported by Bian et al. [13], were adopted from our previous study [18], where their parameters were calibrated against all-atom simulations to ensure accurate representation of their multivalent charge and molecular geometry within the Martini 3 framework. Each particle consists of a gold core (~0.49 nm in diameter) functionalized with TMA ligands. An all-atom model was first constructed and relaxed via simulated annealing, then mapped to a CG representation using the Martini 3 force field [19,20,21]. Four phosphate anions, P2^4−^, P3^3−^, P4^6−^, and P6^6−^, were used as counterions, with parameters calibrated in prior work [18] against all-atom simulations using CGenFF [22,23]. FCC superlattices were assembled by placing four Au–TMA particles in a box, neutralized by one type of phosphate anion, and solvated with water beads.

Simulations were performed in GROMACS 2020 [24] using the Verlet cut-off scheme. Systems were equilibrated for 30 ns, followed by 500 ns production runs with a 20 fs time step. Temperature (300 K) and pressure (1 atm) were maintained using a stochastic velocity rescaling thermostat [25] and Parrinello-Rahman barostat [26], respectively. Short-range interactions used a 1.1 nm cut-off; long-range electrostatics were treated with particle mesh Ewald summation method [27]. Snapshots were visualized with VMD [28,29], and MD data were analyzed using MDAnalysis [30,31]. Spatial regions within the superlattice were defined as follows: tetrahedral cavities (within 2 nm of FCC corners), octahedral cavities (within 2.5 nm of face centers), and the NP–NP interface (remaining void space).

To quantitatively analyze the binding kinetics of anions within the different spatial compartments of the superlattice, we calculated the characteristic average residence time, ⟨*τ*⟩, using the following procedure. For each compartment, continuous residence events were identified as uninterrupted sequences of trajectory frames during which an anion remained inside the compartment. The survival probability, C(t), defined as the fraction of anions from the initial population that had not yet exited the compartment by time *t*, was calculated according to Equation (1):

(1) $$C(t)=\frac{N(t)}{N_{0}}$$

where N(t) is the number of anions still residing in the compartment at time *t*, and is the initial number of anions at *t* = 0.

To capture the kinetics of escape, all residence events were aligned at their start times, and the averaged decay profile was fitted to a double-exponential function (Equation (2)), reflecting multiple binding modes with distinct time constants ($\lambda_{1}$ and $\lambda_{2}$) and amplitudes (*A*_1_ and *A*_2_).

(2) $$C(t)=A_{1}e^{-t\lambda_{1}}+A_{2}e^{-t\lambda_{2}}$$

Finally, the characteristic average residence time ⟨*τ*⟩ was computed from the fitted parameters using Equation (3), which provides an amplitude-weighted mean lifetime that robustly quantifies the overall strength of anion confinement in each compartment under various electric field conditions.

(3) $$\langle \tau \rangle =\frac{A_{1}}{\left(A_{1}+A_{2}\right)\lambda_{1}}+\frac{A_{2}}{\left(A_{1}+A_{2}\right)\lambda_{2}}$$

## 3. Results and Discussion

### 3.1. Electric Field-Dependent Conductivity

**Figure 1:**
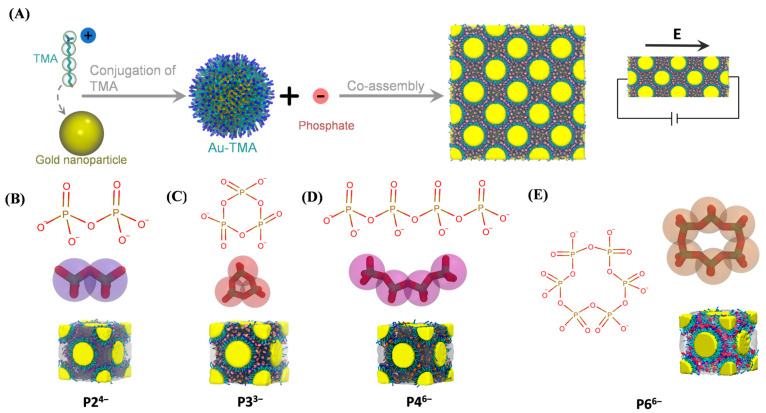
Construction of Au–TMA NP superlattices. (**A**) Schematic illustration of Au–TMA NP formation via conjugation of TMA ligands to gold NPs, followed by electrostatic co-assembly with multivalent phosphate anions into superlattices. The gold core is shown as a yellow sphere; carbon, nitrogen, and sulfur atoms in TMA are colored cyan, blue, and brown, respectively. Hydrogen atoms are omitted for clarity. In the CG model, each TMA ligand is represented by one positively charged bead and three neutral beads. (**B**–**E**) Representative simulation snapshots of Au–TMA superlattices assembled with various phosphate anions: P2^4−^ (**B**), P3^3−^ (**C**), P4^6−^ (**D**), and P6^6−^ (**E**).

We first constructed Au–TMA NPs by decorating gold cores with TMA ligands, following the experimental framework reported by Bian et al. [13] (Figure 1A; see Section 2). The assembly process involves electrostatic co-assembly of Au–TMA NPs with multivalent phosphate anions, yielding FCC superlattices. For MD simulations, four Au–TMA NPs were placed in a simulation box and neutralized with one of four phosphate species—P2^4−^, P3^3−^, P4^6−^, or P6^6−^—to generate representative systems (Figure 1B–E).

**Figure 2:**
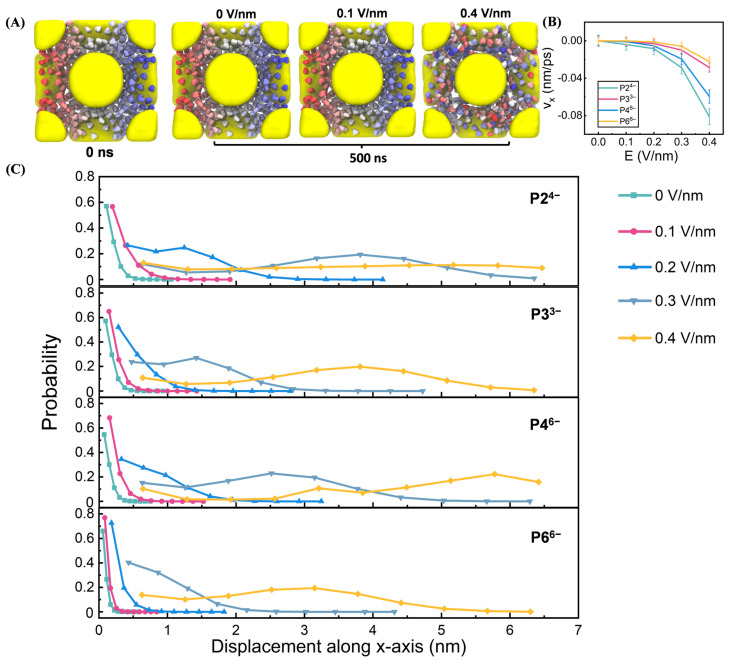
Electric field–driven anion dynamics in Au–TMA NP superlattices. (**A**) Snapshots of the representative Au–TMA/P3^3−^ superlattice at 0 ns (pre-field reference) and after 500 ns under varying electric field strengths. P3^3−^ anions are color-coded along the x-axis from red (left) to blue (right) to visualize spatial displacement. (**B**) Average anion velocities along the x-axis for the four NP superlattices under increasing electric field strengths. (**C**) Displacement distributions of anions along the x-axis for each superlattice system under the same field conditions as in (**B**).

Following 30 ns equilibration, uniform electric fields (0–0.4 V/nm) were applied along the x-axis (Figure 2A). The electric field strength was varied from 0 to 0.4 V/nm. This upper limit was selected to achieve a clear resolution of the field-gated conductive transition and the anion-dependent mobility trends within computationally feasible simulation times, while ensuring the structural integrity of the soft-matter superlattice. Fields significantly exceeding this value were found to risk irreversible deformation of the electrostatic assembly, shifting the focus from ion transport within a stable framework to material breakdown. The chosen range successfully captures the critical non-linear response regime relevant to operational conditions in nanoconfined systems, where local field enhancements are significant, without compromising the system’s stability.

Visual inspection of the P3^3−^ system revealed enhanced anion dispersion and migration under strong fields (e.g., 0.4 V/nm), in contrast to more localized anion distributions at 0.1 V/nm. To quantify field-driven ion dynamics, we calculated anion velocities along the field direction (Figure S1), with averages presented in Figure 2B. Anion mobility followed the order P2^4−^ > P4^6−^ > P3^3−^ > P6^6−^, suggesting that both charge density and geometry modulate transport. Anion displacements along the electric field further supported this trend (Figure 2C), with differences becoming more pronounced at higher field strengths (≥0.3 V/nm). The consistency between displacement and velocity data reinforces the anion-dependent nature of electric field–driven transport.

**Figure 3:**
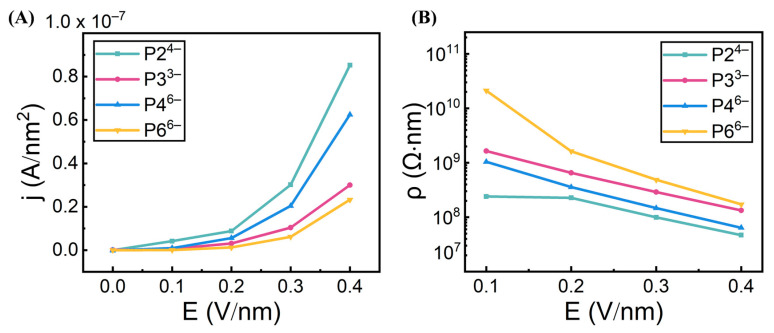
Field-dependent ionic conductivity in Au–TMA NP superlattices. (**A**) Ionic currents calculated from anion velocities in the four superlattice systems under varying electric field strengths. (**B**) Corresponding resistivity values of each superlattice, showing an inverse trend with ionic current across different field conditions.

We next computed ionic currents based on anion velocities (Figure 3A), which mirrored the observed trends. The corresponding resistivity values (Figure 3B) exhibited the expected inverse relationship, confirming that ionic conductivity increases under strong electric fields. All systems remained low conductivity at 0.1–0.2 V/nm, but exhibited a marked rise in conductivity at 0.3–0.4 V/nm, consistent with the semiconducting, field-gated behavior recently reported for citrate–AuNP superlattices by Lionello et al. [16] Importantly, our results not only demonstrate this electrostatic-gating semiconductor-like behavior, but also reveal the underlying mechanism—how anion-specific properties, such as size, charge density, and geometry, govern this transition. For instance, P2^4−^ outperforms P3^3−^ despite similar size, while P4^6−^ exhibits higher conductivity than the smaller P3^3−^, highlighting charge density as a more dominant factor than size alone. Furthermore, linear anions (P2^4−^, P4^6−^) consistently display greater conductivity than their ring-shaped counterparts (P3^3−^, P6^6−^). This difference is attributed to the bulkier size and stronger interfacial adsorption of cyclic anions (likely due to enhanced vdW interactions from a larger contact area), which restricts their mobility under electric fields.

The observed hierarchy in conductivity (P2^4−^ > P4^6−^ > P3^3−^ > P6^6−^) arises from the interplay between the electrophoretic driving force and the interactions of anions with the superlattice framework. The electrophoretic force, proportional to anion charge (F = qE), is the primary determinant of mobility, explaining why P4^6−^ (charge: −6) outperforms the smaller P3^3−^ (charge: −3), and why P2^4−^ (charge: −4), with its high charge-to-size ratio, is the most effective carrier. Mobility is counteracted by two key factors: (i) steric hindrance, where bulky, rigid rings of P3^3−^ and P6^6−^ impede passage through confined interstitial spaces, and (ii) interfacial adsorption, where delocalized charges and larger contact areas of cyclic anions lead to stronger van der Waals and electrostatic interactions with the TMA-functionalized NP surfaces, effectively trapping them. The superior performance of linear anions thus reflects their ability to combine a high electrophoretic drive with minimal steric and adsorptive retardation.

### 3.2. Compartment-Specific Anion Dynamics

**Figure 4:**
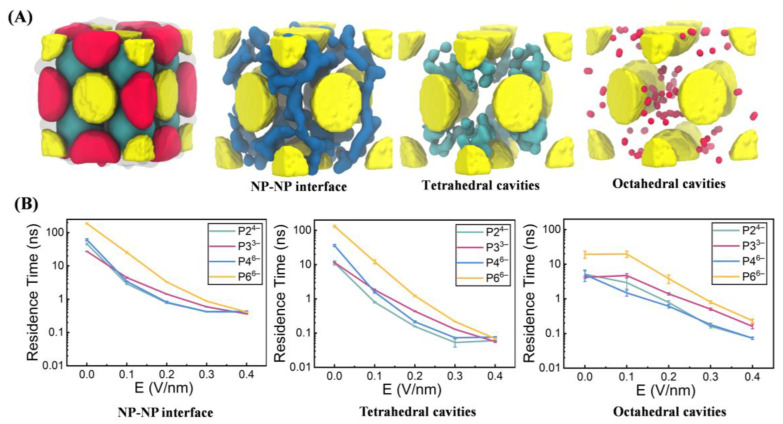
Spatial distribution and residence dynamics of phosphate anions in NP superlattices. (**A**) Spatial segmentation of anion occupancy within the NP superlattice unit cell into three compartments: NP–NP interfaces (transparent surface), tetrahedral cavities (cyan surface), and octahedral cavities (red surface). The left panel depicts the overall partitioning, while the right panels show representative anions in each region, colored blue, cyan, and red, respectively. (**B**) Mean residence times of anions within the three compartments as a function of applied electric field strength, illustrating the modulation of anion mobility and spatial confinement by external fields.

To further understand anion mobility within the NP superlattice, we analyzed phosphate ions’ spatial dynamics across three spatial compartments: the NP–NP interface, tetrahedral cavities, and octahedral cavities (Figure 4A), following the compartmentalization strategy described by Lionello et al. [16] (see Section 2). To quantify the kinetics of anion binding within each compartment, we calculated the residence time using Equations (1)–(3) (see Section 2). Briefly, for each anion in a specific compartment (e.g., the NP-NP interface), we tracked its continuous occupancy and constructed a survival profile of the fraction of anions, C(t), that remained in the compartment over time (Figure S2). This decay profile was fitted to a double-exponential function (Equation (2)) to capture multiple binding modes, and the characteristic average residence time ⟨τ⟩ was obtained using Equation (3). The resulting ⟨τ⟩ values across all compartments and electric field strengths are shown in Figure 4B, providing a direct quantification of the trapping strength of each region.

In the absence of an external electric field, anions exhibited the longest residence times at the NP–NP interfaces, followed by the tetrahedral and octahedral cavities (Figure 4B). This hierarchy reflects stronger electrostatic adsorption and greater steric confinement at the interparticle contact regions. Upon application of increasing field strengths (≥0.2 V/nm), residence times across the three compartments progressively converged, indicating enhanced anion mobility and accelerated intercompartmental exchange under electric driving forces. Notably, across all field conditions, anion mobility consistently followed the order P2^4−^ > P4^6−^ > P3^3−^ > P6^6−^. This trend aligns with the conductivity profiles shown in Figure 3 and highlights the combined influence of molecular size, geometry, and charge density—where reduced size and higher charge density promote more efficient transport within the superlattice framework.

**Figure 5:**
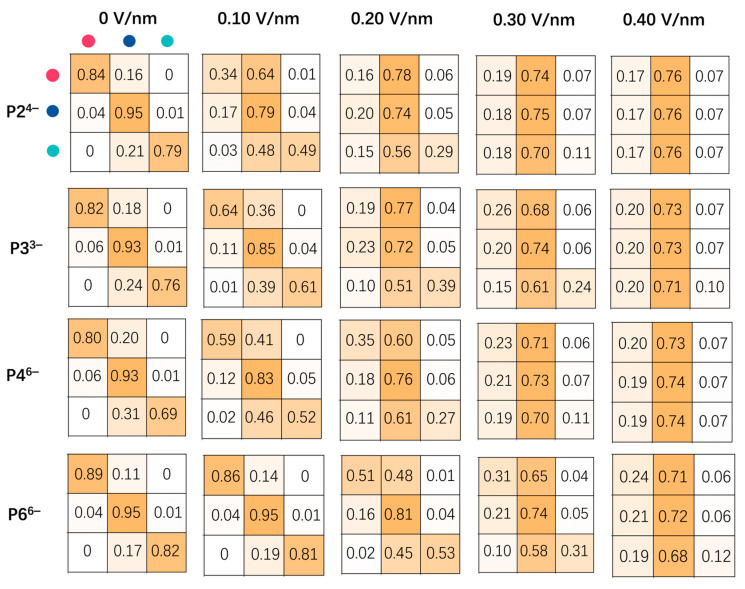
Transition probabilities of phosphate anions among superlattice compartments under varying electric fields. Transition matrices depict the probabilities of anions migrating among three distinct regions within the NP superlattice: octahedral cavities (red spheres), NP–NP interfaces (blue spheres), and tetrahedral cavities (cyan spheres). Each row corresponds to the origin compartment, with the sum of probabilities in each row normalized to unity, reflecting the likelihood of anion transitions under different applied electric field strengths. Color intensity ranges from white (probability = 0) to orange (probability = 1).

To elucidate internal barriers to phosphate ion transport, we analyzed anion migration pathways by computing transition probabilities among the three defined compartments—NP–NP interfaces, tetrahedral cavities, and octahedral cavities—under varying electric field strengths (Figure 5). This analysis provides a systematic comparison across four phosphate anions (P2^4−^, P3^3−^, P4^6−^, and P6^6−^) and field strengths ranging from 0 to 0.40 V/nm. For each anion, the resulting transition matrix quantifies the likelihood of migration from one compartment to any of the three, with row-wise probabilities normalized to unity.

At 0 V/nm, all anions predominantly remained confined within their initial compartments, as reflected by diagonal probabilities exceeding 0.8, indicating minimal spontaneous redistribution (Figure 5). Upon application of stronger electric fields (≥0.2 V/nm), transitions to the NP–NP interface became markedly more frequent for P2^4−^, P3^3−^, and P4^6−^, with interface occupation probabilities rising to ~0.7, highlighting increased field-induced localization at interparticle contact zones. In contrast, P6^6−^ retained higher probabilities of remaining in the tetrahedral and octahedral cavities even under strong fields, likely due to its bulky, ring-like geometry and delocalized charge, which hinder its lateral mobility. These results underscore the interplay between anion structure and external fields in regulating spatial dynamics within NP superlattices.

Interestingly, across all systems including P6^6−^, increased transition flux toward the NP–NP interface was often followed by subsequent redistribution toward octahedral and tetrahedral cavities. This preference reflects the interplay between adsorption strength and geometric confinement: the NP–NP interface imposes strong electrostatic binding and steric hindrance, while the octahedral and tetrahedral cavities offer lower adsorption energies and reduced confinement. The deviation observed in the P6^6−^ system—characterized by limited intercompartmental mobility—can be attributed to its bulky ring-shaped structure and enhanced interfacial affinity, which require higher field strengths to overcome.

Together, these results demonstrate the critical influence of anion structure on compartment-specific dynamics within NP superlattices. Linear anions (e.g., P2^4−^) exhibit enhanced mobility under electric fields, facilitating dynamic redistribution across compartments. In contrast, ring-shaped anions (e.g., P6^6−^) are prone to adsorption and resist field-driven reconfiguration. These findings suggest the potential of anion engineering to modulate charge transport and spatial organization in NP superlattices, with implications for optimizing performance in electrochemical sensing and ion transport applications.

### 3.3. Field-Driven Anion Redistribution

**Figure 6:**
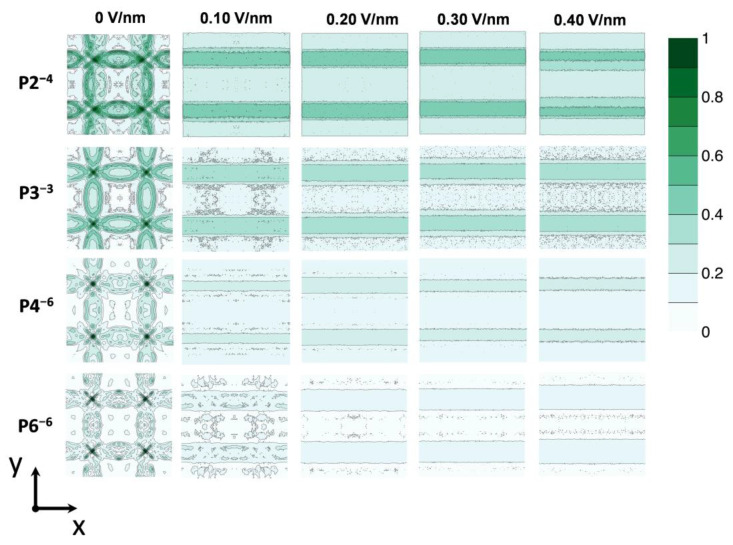
Anion density distributions on the X-Y plane under varying applied electric fields. Firstly, anion density maps within the superlattices were counted with three-dimensional arrays. Specifically, geometric center of each anion was obtained and classified into the density arrays. Then, to facilitate the visualization of the anion densities within superlattices, three-dimensional arrays of anion densities were further projected onto the X-Y plane by summing density arrays along Z-axis, which generated two-dimensional density arrays. Finally, the two-dimensional density arrays were then further normalized by dividing the maximum of all two-dimensional density arrays. Density patterns vary systematically with increasing electric field strength, illustrating field- and anion-dependent migration and accumulation behaviors.

To further elucidate the accumulation of anions at NP–NP interfaces under applied electric fields, we mapped the overall anion density distribution within the NP superlattice by projecting ion positions onto the X-Y plane (see Figure 2A) and normalizing to generate density profiles (Figure 6). The spatial distribution is influenced by both the electric field strength (0 to 0.40 V/nm) and anion characteristics. At 0 V/nm, anions remain evenly dispersed around Au–TMA NPs, reflecting the absence of directional driving forces. Increasing field strength induces progressive migration toward NP–NP interfaces and tetrahedral cavities, with species such as P2^4−^, P3^3−^, and P4^6−^ redistributing at relatively low fields (0.1–0.2 V/nm), whereas P6^6−^ requires stronger fields for comparable relocation, emphasizing the role of size and geometry in modulating mobility.

Anion charge also critically affects field response: higher-charged anions (e.g., P6^6−^) exhibit more pronounced spatial gradients than lower-charged counterparts like P3^3−^. For example, at 0.40 V/nm, P6^6−^ concentrates sharply at one edge of the plane, while P3^3−^ shows a more gradual density gradient, reflecting weaker electrophoretic driving forces. This charge-dependent redistribution aligns with classical transport theory, such as the Nernst-Planck equation, where ion flux arises from the balance of electromigration and diffusion. Collectively, these findings reveal that the electric field magnitudes and ion properties (i.e., charges, sizes and shapes) jointly govern the dynamic spatial organization of anions in NP superlattices.

The anion density maps in Figure 6 reveal a classic competition between electromigration and adsorption, which can be quantitatively interpreted using models such as the Nernst-Planck equation. At low fields, thermal motion and entropic effects dominate, leading to relatively uniform anion distributions. As the field increases, the electrophoretic force drives anions against the electrostatic potential wells created by positively charged NP interfaces, resulting in accumulation at the NP–NP interfaces—a phenomenon akin to electrostatic trapping in the Stern layer. The pronounced polarization of P6^6−^ at high fields, compared to the more gradual gradient of P3^3−^, reflects both its higher charge, yielding stronger electrophoretic drive (F = qE), and stronger adsorption, which necessitates a higher field to overcome the activation barrier for desorption and migration. This dynamic, in which anions are continuously adsorbed and field-desorbed, underpins the net ionic current through the superlattice.

## 4. Broader Implications and Future Prospects

The electric fields considered in this work (0–0.4 V/nm) are higher than those typically applied in bulk experiments, but were chosen to overcome the intrinsic timescale limitations of MD simulations. It is noteworthy that local fields in nanoconfined environments—such as superlattice interstices or near charged interfaces—can be strongly amplified, reaching magnitudes comparable to those used here [32]. Consequently, the mechanistic trends revealed in this study, including the enhanced mobility of linear anions and the critical role of charge density, are expected to remain valid under experimentally realizable fields, albeit with shifted thresholds and slower kinetics.

These principles of selective ion transport, governed by molecular geometry and nanoconfinement, have direct and profound implications for biological systems. For instance, our findings provide a physical blueprint for the design of synthetic biomimetic ion channels. The ability to discriminate between anions based on shape and charge density, as demonstrated with linear and cyclic polyphosphates, could be engineered into nanoparticle-based membranes to selectively filter biologically relevant anions like chloride, bicarbonate, or ATP, with potential applications in targeted drug delivery or metabolic sensing.

This geometric distinction is inherent to key biological molecules. The phosphate groups in the backbones of DNA and RNA are structural analogs to the anions in our study. Our observation that linear polyphosphates exhibit higher mobility and distinct binding compared to cyclic forms offers a physical basis for understanding the dynamics of nucleic acids and their interactions with proteins. Extending this concept to metabolites, the stark geometric contrast between linear molecules like ATP and cyclic signaling molecules like cAMP suggests that their inherent shape and charge distribution are critical factors influencing their diffusion and interaction kinetics within crowded cellular environments, such as biomolecular condensates.

From a translational perspective, the field-gated semiconductor-like behavior we observed positions these superlattices as promising candidates for a new class of bioelectronic devices. Functionalizing the nanoparticles with specific receptors could allow a target biomolecule (e.g., a protein or virus) to bind within the interstitial spaces, thereby modulating the ionic conductivity and generating an amplified electrical signal for label-free detection. The biocompatibility of gold nanoparticles and the aqueous environment of our simulations further support the potential for developing direct neural interfaces or other bio-integrated sensors.

Looking forward, future investigations will explicitly incorporate biological complexity to bridge the gap between inorganic nanomaterials and physiology. This includes simulating superlattices in biological fluids, studying competitive binding with proteins, and designing systems responsive to physiological stimuli like pH changes. By elucidating these fundamental structure-transport relationships, our work lays the foundation for designing intelligent, bio-integrated materials for advanced diagnostics, therapeutics, and fundamental biophysical research.

## 5. Conclusions

Our simulations reveal that anion identity, specifically geometry and charge density, plays a central role in governing electric field–induced transport in NP superlattices. Linear anions with high charge density exhibit markedly enhanced mobility and contribute to elevated ionic conductivity, whereas ring-shaped counterparts with delocalized charge demonstrate restricted diffusion due to stronger interfacial adsorption and geometric rigidity. Among the structural descriptors examined, charge density consistently exerts a greater influence than ionic size on transport efficiency.

Specifically, elevated voltages lead to a concentration of ion migration pathways into preferential transport “conduits” (Figure 6). Unless physically obstructed by gold nanoparticles (NPs), ions tend to migrate predominantly along these low-resistance “conduits” under high field strengths. Compartment-specific analyses reveal that while the NP-NP interfaces ultimately accumulate the highest anion density (with the longest residence times), anions traverse the tetrahedral cavities, characterized by the weakest constraints and the greatest ease. Transition probability matrices further corroborate that linear anions, benefiting from their conformational flexibility, overcome geometric confinement more readily under increasing field strengths, particularly when navigating through less constrained regions like tetrahedral cavities and “conduits”, demonstrating a greater propensity for “escape”. Conversely, cyclic species, due to their rigid structures, display more pronounced kinetic trapping within regions of stronger confinement, such as octahedral compartments and NP-NP interfaces. These field-dependent migration patterns are supported by spatial density maps, which exhibit a marked polarization drive of anions.

Together, these findings establish anion geometry and charge density as key tunable parameters for directing ionic transport in NP superlattices. This work provides fundamental insights into ion–NP interactions under external fields and opens up new opportunities for the rational design of responsive superlattice-based materials for applications in ionic electronics, electrochemical sensing, and energy storage. Future investigations incorporating explicit solvent models, mixed-valency systems, or dynamic electrode interfaces may further enhance the predictive control of ionic conductivity in these emerging nanostructured platforms.

## Data Availability

The original contributions presented in this study are included in the article/Supplementary Materials. Further inquiries can be directed to the corresponding author.

## Supplementary Materials

The following supporting information can be downloaded at: https://www.mdpi.com/article/10.3390/biom15101427/s1, Figure S1: Velocity profiles of anions along the external field direction; Figure S2: Time-dependent decay of phosphate anion residence within distinct compartments.
