# Supplementary material for: Field-Gated Anion Transport in Nanoparticle Superlattices Controlled by Charge Density and Ion Geometry: Insights from Molecular Dynamics Simulations
Source: Biomolecules. 2025 Oct 8;15(10):1427. doi: 10.3390/biom15101427 (PMC12563655; doi:10.3390/biom15101427)
Supplement: Supplementary file 1 [file biomolecules-15-01427-s001.zip › biomolecules-3886509-supplementary.pdf]

Supplementary Information for

Field-Gated Anion Transport in Nanoparticle Superlattices Controlled by Charge Density and Ion Geometry: Insights from Molecular Dynamics Simulations

Yuexin Su [1], Jianxiang Huang [2], Zaixing Yang [2], Yangwei Jiang [1] and Ruhong Zhou [1,2,3,*]

1 School of Physics, Zhejiang University, Hangzhou 310027, China

2 Institute of Quantitative Biology, College of Life Sciences, Zhejiang University, Hangzhou 310027, China

3 Department of Chemistry, Columbia University, New York, NY10027, USA

* Correspondence: rhzhou@zju.edu.cn

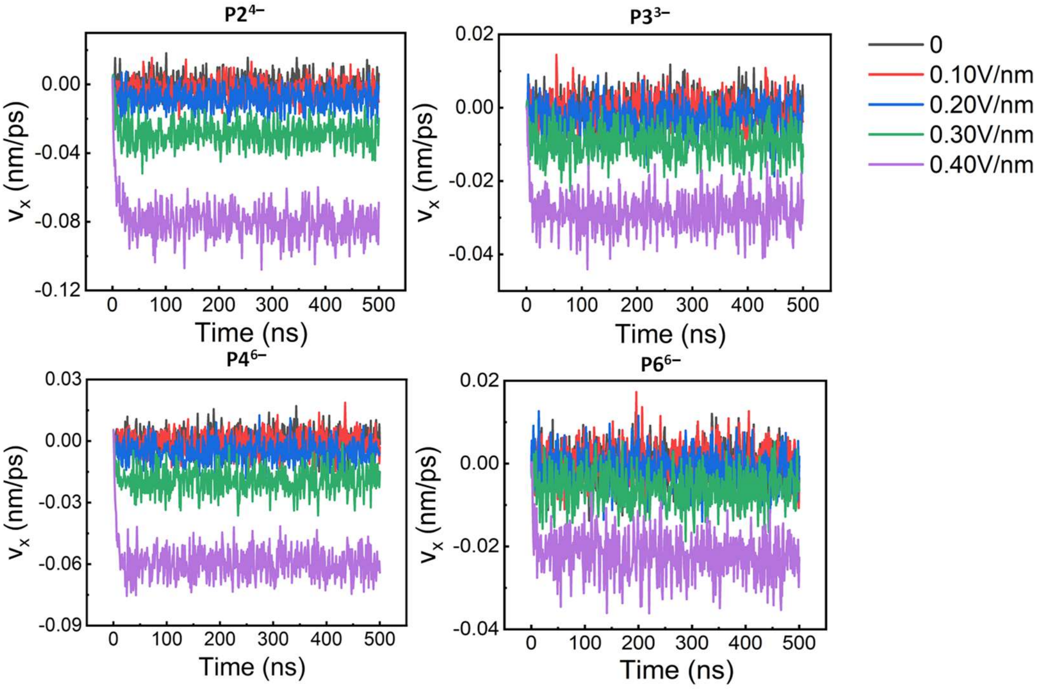

Figure S1. Velocity profiles of anions along the external field direction. The figure shows the distribution of anion velocities projected along the direction of the applied electric field, providing insights into their directional mobility under field-driven conditions.

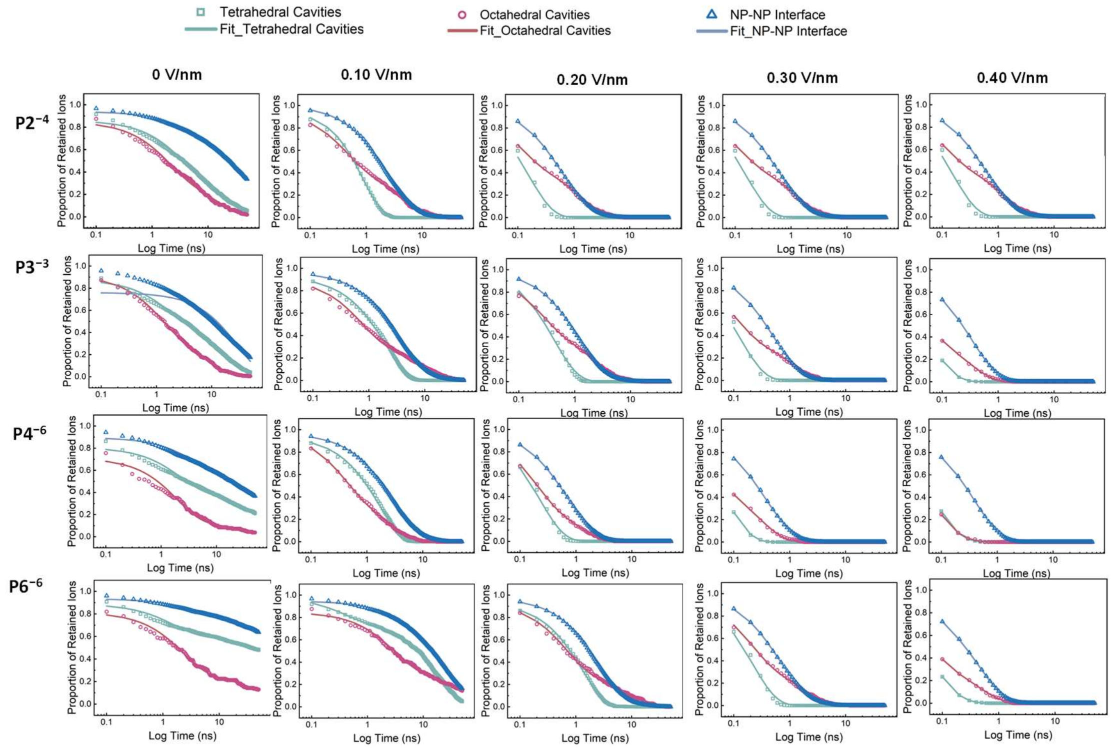

Figure S2. Time-dependent decay of phosphate anion residence within distinct compartments. Residence time distributions were fitted using double-exponential decay functions (solid lines), capturing the heterogeneous retention dynamics of phosphate anions across different microenvironments.
